# Systematic bibliometric and visualized analysis of research hotspots and trends on the application of artificial intelligence in diabetic retinopathy

**DOI:** 10.3389/fendo.2022.1036426

**Published:** 2022-10-31

**Authors:** Ruoyu Wang, Guangxi Zuo, Kunke Li, Wangting Li, Zhiqiang Xuan, Yongzhao Han, Weihua Yang

**Affiliations:** ^1^ The Fourth School of Clinical Medicine, Nanjing Medical University, Nanjing, China; ^2^ The First School of Clinical Medicine, Nanjing Medical University, Nanjing, China; ^3^ Shenzhen Eye Hospital, Jinan University, Shenzhen, China; ^4^ Institute of Occupational Health and Radiation Protection, Zhejiang Provincial Center for Disease Control and Prevention, Hangzhou, China; ^5^ Affiliated Jiangning Hospital, Nanjing Medical University, Nanjing, China

**Keywords:** artificial intelligence, diabetic retinopathy, bibliometric, CiteSpace, systematic analysis

## Abstract

**Background:**

Artificial intelligence (AI), which has been used to diagnose diabetic retinopathy (DR), may impact future medical and ophthalmic practices. Therefore, this study explored AI’s general applications and research frontiers in the detection and gradation of DR.

**Methods:**

Citation data were obtained from the Web of Science Core Collection database (WoSCC) to assess the application of AI in diagnosing DR in the literature published from January 1, 2012, to June 30, 2022. These data were processed by CiteSpace 6.1.R3 software.

**Results:**

Overall, 858 publications from 77 countries and regions were examined, with the United States considered the leading country in this domain. The largest cluster labeled “automated detection” was employed in the generating stage from 2007 to 2014. The burst keywords from 2020 to 2022 were artificial intelligence and transfer learning.

**Conclusion:**

Initial research focused on the study of intelligent algorithms used to localize or recognize lesions on fundus images to assist in diagnosing DR. Presently, the focus of research has changed from upgrading the accuracy and efficiency of DR lesion detection and classification to research on DR diagnostic systems. However, further studies on DR and computer engineering are required.

## Introduction

Diabetic retinopathy (DR) is a complication of diabetes that affects the fundus blood vessels. It poses a major threat to vision loss and blindness in patients with diabetes (1). In 2015, approximately 415 million people worldwide were diagnosed with diabetes. This number is expected to increase to 642 million by 2040 (2). It is estimated that more than one-third of people with diabetes worldwide have some form of DR, and approximately one-tenth of people with diabetes have vision-threatening DR, including proliferative DR and diabetic macular edema (3). Regular screening is crucial for patients with DR; however, traditional detection methods are inadequate for many patients. Traditional screening or diagnostic methods require professional doctors and are time-consuming, laborious, and expensive. As a result, large-scale early screening remains a significant challenge.

With the emergence and continuous development of artificial intelligence, the integration of AI in healthcare, including screening of major ophthalmic diseases, becomes a research hotspot. AI refers to the use of computers to simulate intelligent behavior with little or no human intervention, which is a broad term (4). This technology involves many aspects, including traditional machine learning (ML) and deep learning (DL) (5). In the phase of traditional ML, through the feature extraction, AI can localize lesions on retinal images to diagnose and grade DR based on imaging biomarkers, including microaneurysms, hard exudation, cotton-wool spots, and macular edema. With the emergence of DL, which uses multilevel and multineuron learning algorithms, several system using DL for DR screening has been constructed and achieved higher specificity and sensitivity than the ones using traditional ML (6, 7). As AI technology continuously developing, more and more AI research is combining knowledge-driven and data-driven approaches, which is also reflected in DR research (8). The integration of AI in DR screening will significantly improve diagnostic efficiency, save workforce and financial resources, and make remote diagnosis possible in remote and poor areas, which is a very promising field.

Bibliometric studies have already been conducted on the AI application in ophthalmic diseases (9–11). Considering the current research situation, an updated bibliometric study on the application of AI in DR was decided to be conducted based on the present research results. This study aimed to use bibliometric methods to analyze the literature retrieved from the Web of Science Core Collection (WoSCC) to assess the global status of the application of AI in DR research and analyze the hotspots and trends. For instance, the scientific citation index literature on AI’s application in DR screening and diagnosis was analyzed using bibliometric methods. The analysis highlighted data relevant to countries and regions, institutions, journals, research categories, keywords, and references. A fundamental objective of our research is to develop a repeatable and unbiased strategy to explore the dynamic frontiers of knowledge in research field. Therefore, active areas, future development areas, and potential barriers to the application of AI in the screening and diagnosis of DR were particularly examined in this study. This report aimed to provide resources for AI professionals, ophthalmologists, diagnostic physicians, and medical imaging researchers.

## Materials and methods

On October 3, 2022, all citation data published between January 1, 2012, and June 30, 2022 were downloaded from WoSCC. Two authors (Guangxi Zuo and Ruoyu Wang) verified the data independently. The retrieval formula was TS = (AI or “Artificial Intelligence” or “neural network” or “transfer learning” or “Machine Learning” or “Deep Learning” or automat* or algorithm) AND TS = (“diabetic eye disease” or “diabetic retinopathy” or “diabetic macular edema”). The search selected English literature and articles and excluded early access, proceedings papers, book chapters, data papers, and retracted publications.

**Figure 1:**
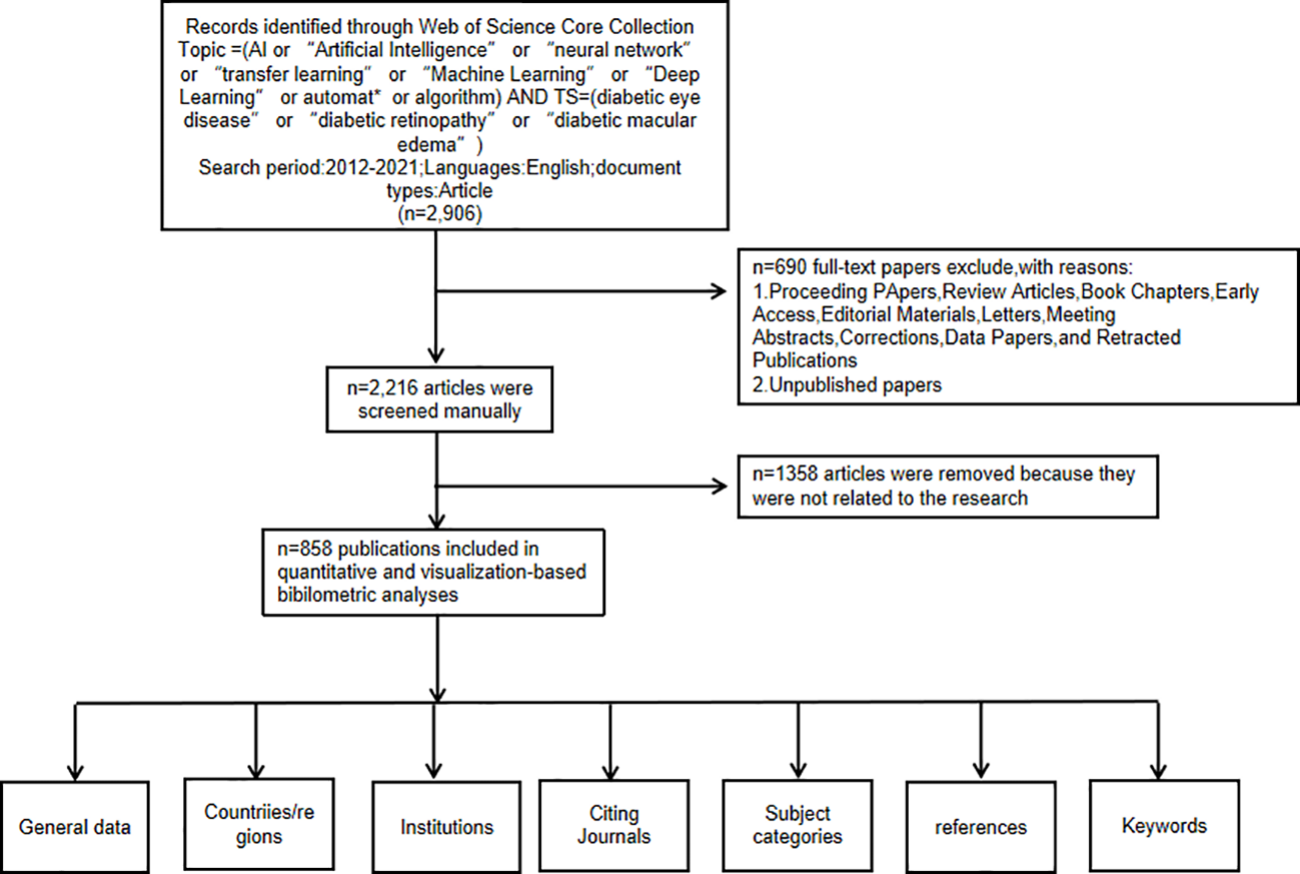
Frame flow diagram showing the detailed selection criteria and bibliometric analysis steps for the study of the application of artificial intelligence in diabetic retinopathy.

To ensure accuracy of the study, the data were manually expurgated after reading the titles and abstracts of study without exception to obtain the most accurate analysis results. The criteria for manual exclusion were as follows:1) the research subject was not DR, and 2) the research method excluded AI. Details of the filtering process are shown in **Figure 1**. The data included in this study were all about the application of AI in detecting and grading DR. We analyzed general data, countries or regions, institutions, citing journals, subject categories, references, and keywords using CiteSpace 6.1.R3. All citation features have been included in this article.

## Results

### Distribution of articles by publication year

We analyzed 858 studies published between January 1, 2012, and June 30, 2022, focusing on the application of AI in detecting and grading DR. The data collected and counted from WoSCC were de-duplicated by means of the duplication removal function of the CiteSpace software to confirm the number of verified data.

**Figure 2:**
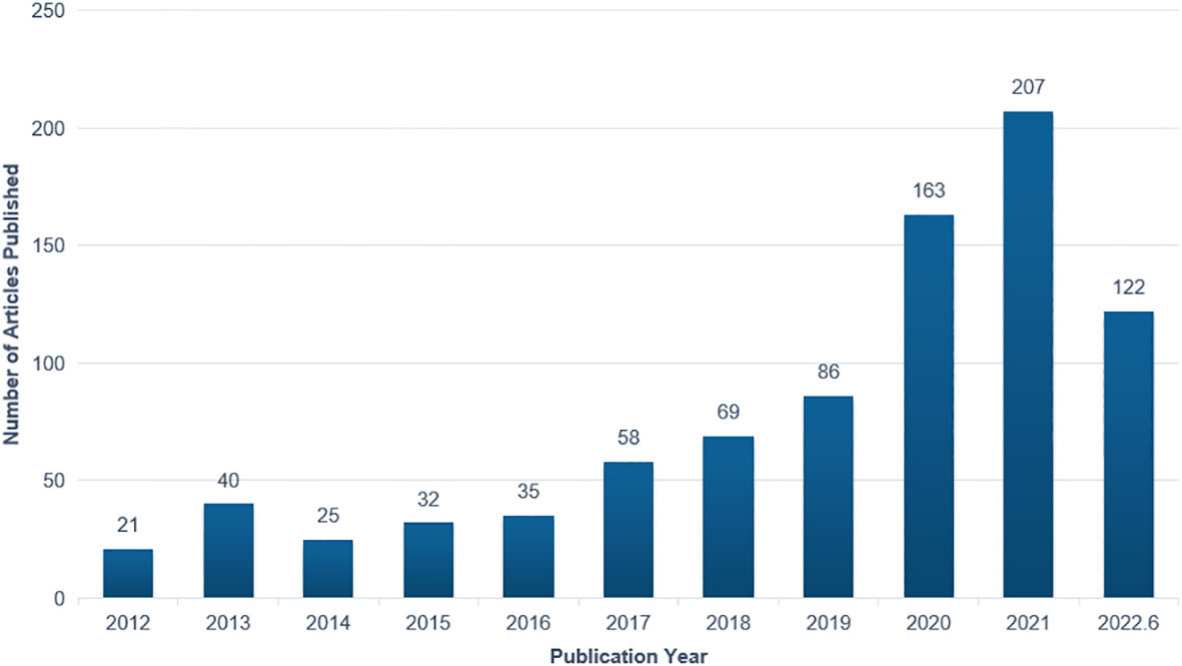
Annual number of publications on the application of artificial intelligence in diabetic retinopathy from 2012 to June 2022.

The annual number of articles on the application of AI in detecting and grading DR increased steadily between 2012 and 2019. It began to rise in 2020 and exceeded 200 for the first time in 2021. The number of publications per year is shown in **Figure 2**.

### Countries or regions

**Figure 3:**
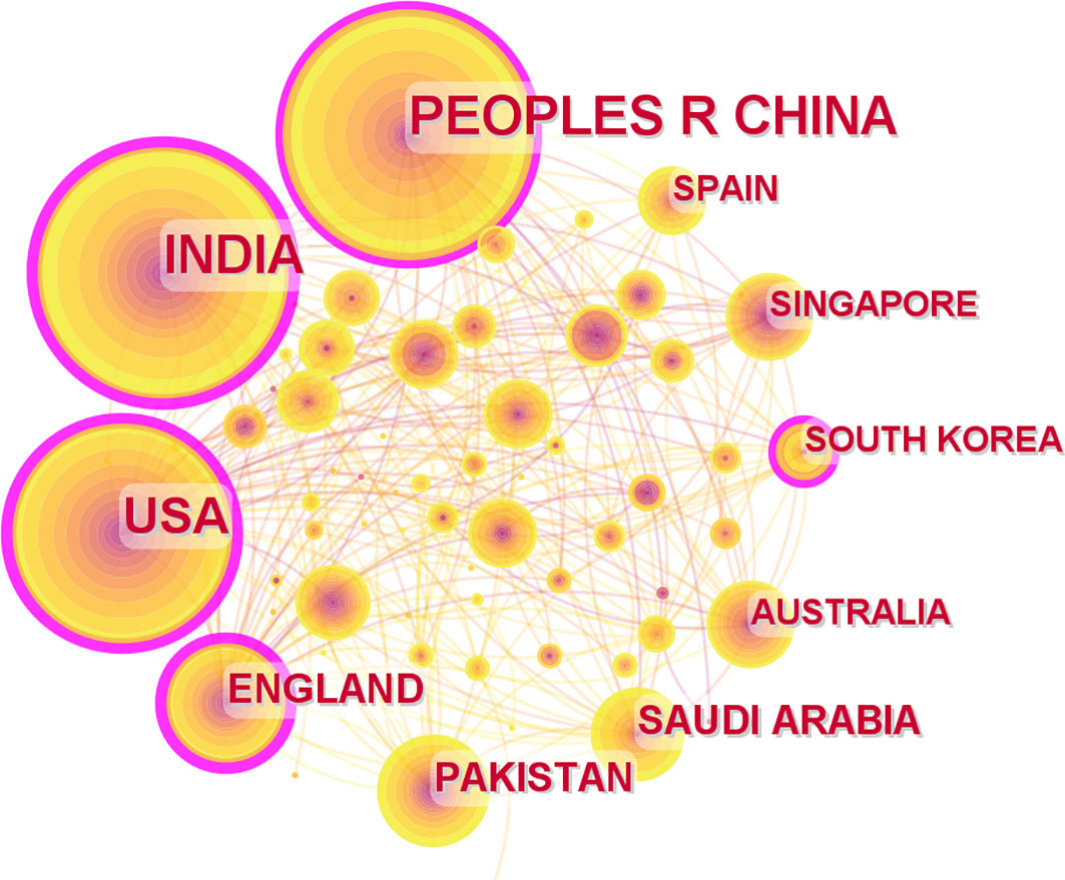
Cooperation of countries or regions that contributed to publications on the use of artificial intelligence for diagnosing diabetic retinopathy from 2012 to 2022.

The CiteSpace software’s default settings were used to count the number of papers in each country and to analyze the cooperation relationships between countries and regions. Overall, the citations involved 77 countries and regions. The size of each label and the yellow node area in **Figure 3** represent the number of citations. The top three countries with large yellow nodes were China, India, and the United States, with 232, 216, and 139 articles, respectively. The cooperative relationships between countries were represented by the links between nodes. Countries with more links are more influential. The influence of a country is illustrated in **Figure 3** by the dimension of the purple circle. Notably, the purple circle corresponding to the “United States” label has the largest area. (0.35), implying that articles published in the United States have the greatest overall influence in the field of AI application in detecting and grading DR. The data in **Table 1** confirm this conclusion. The H-index can precisely reflect a country’s academic achievement (12). The more links a country has, the higher its centrality and the wider the purple circle. Generally, China has the highest publication count, while the United States is regarded as the most influential country.

**Table 1:** Top 10 countries or regions with publications on the application of AI in DR from 2012 to June 2022.

Rank	Countries or regions	Counts	Centrality	H-index
1	China	232	0.20	35
2	India	216	0.23	29
3	United States	139	0.35	42
4	England	59	0.30	21
5	Saudi Arabia	47	0.10	14
6	Pakistan	47	0.06	18
7	Australia	33	0.07	16
8	South Korea	32	0.11	12
9	Singapore	32	0.03	19
10	Spain	30	0.05	11

AI, artificial intelligence; DR, diabetic retinopathy.

### Institutions

**Figure 4:**
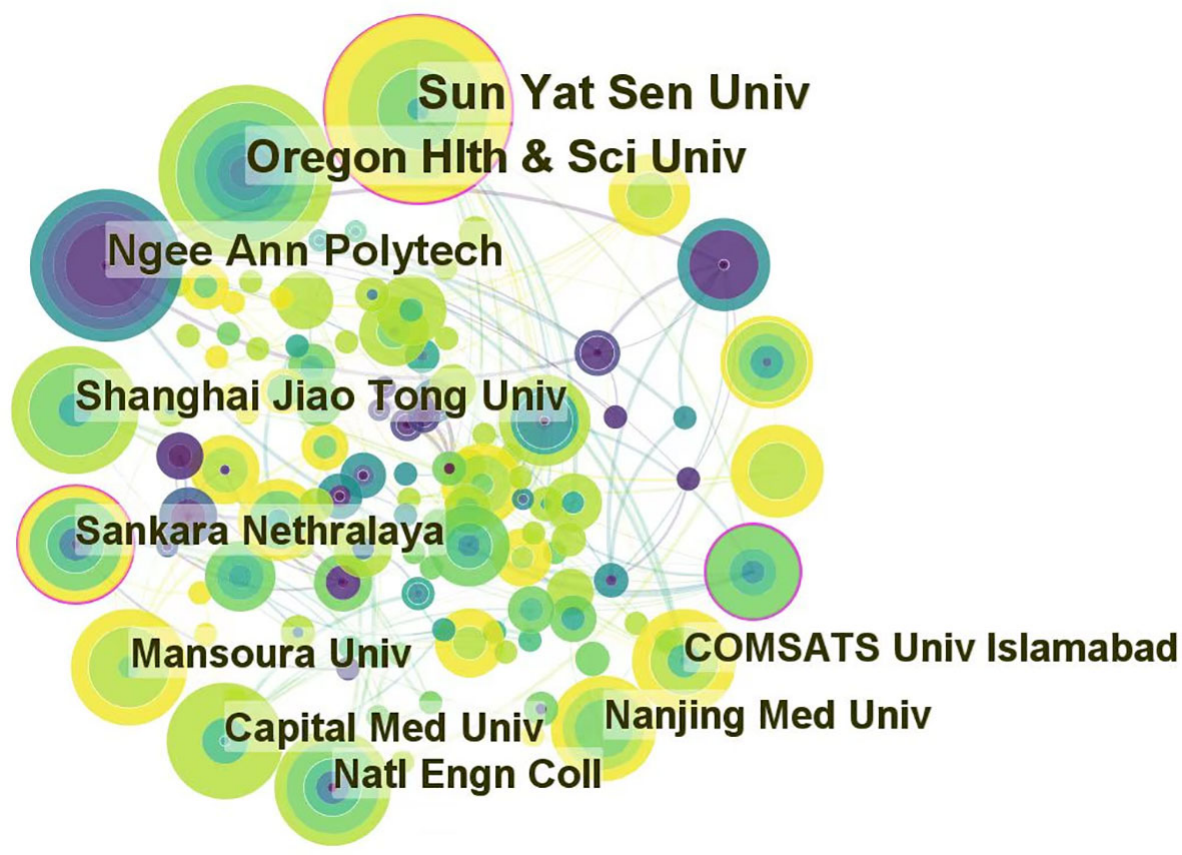
Cooperation of institutions that contributed to publications on the use of artificial intelligence in diabetic retinopathy from 2012 to June 2022.

The top 10 institutions of the selected articles focusing on the application of AI in detecting and grading DR are presented in **Table 2**. The data displayed are the results of CiteSpace’s default setting. However, 5 out of 10 are Chinese institutions because China has the maximum sum of publications. Institutions in the United States, Singapore, India, Egypt, and Pakistan were included in addition to Chinese institutions. The size of the nodes in **Figure 4** is positively correlated with the number of articles published by each institution. Furthermore, the links between labels reflect cooperative relationships among institutions. Regarding academic achievements, the United States and a Pakistanian institution had the highest H-index. Two of the top five institutions with the highest H-indices were Chinese institutions.

**Table 2:** Top 10 institutions with publications on the application or the use of AI in DR from 2012 to June 2022.

Rank	Institution	Counts	H-index	Countries or regions
1	Sun Yat-Sen University	16	7	China
2	Oregon Health and Science University	15	10	United States
3	Ngee Ann Polytech	13	9	Singapore
4	Shanghai Jiao Tong University	11	7	China
5	Mansoura University	10	8	Egypt
6	Sankara Nethralaya	10	7	India
7	Capital Medical University	10	7	China
8	National Engineering College	10	6	India
9	**COMSATS University Islamabad**	9	8	Pakistan
10	Nanjing Medical University	9	5	China

AI, artificial intelligence; DR, diabetic retinopathy.

### Journals and research category

**Figure 5:**
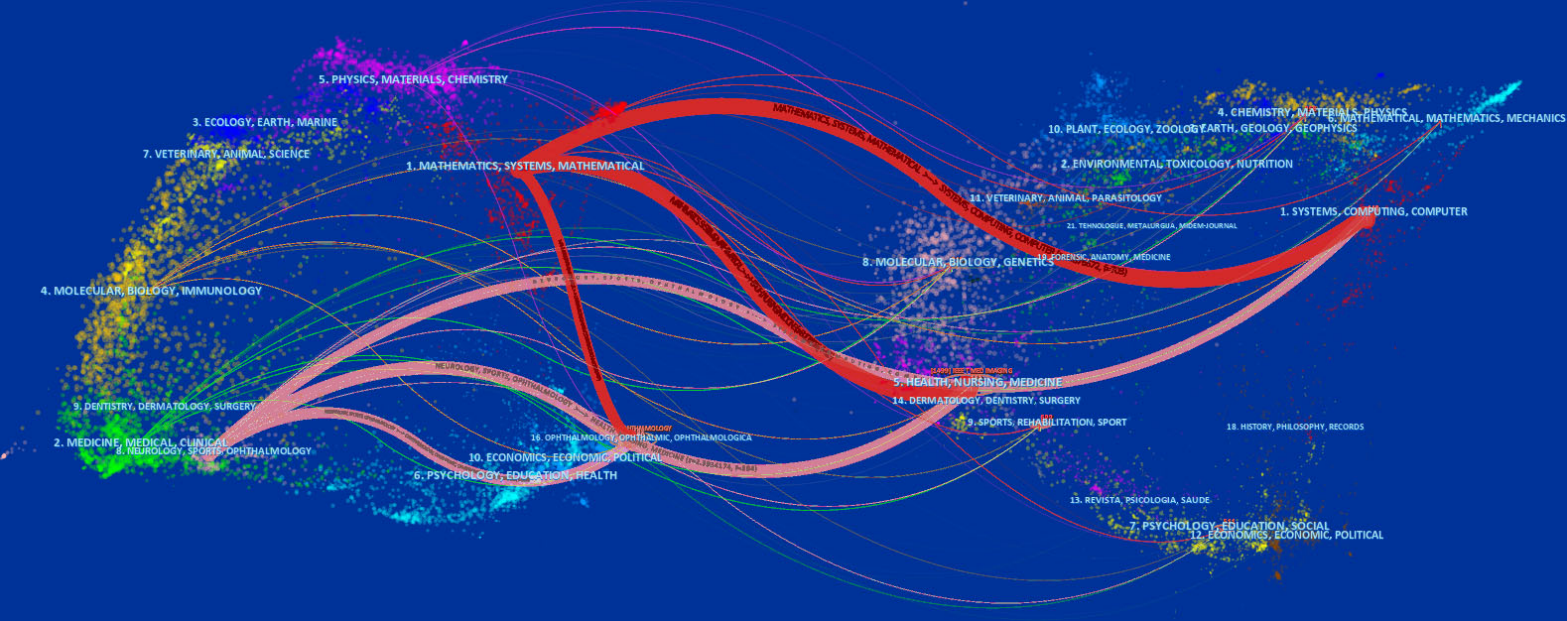
Dual map overlay of journals that contributed to publications on the use of artificial intelligence in diabetic retinopathy from 2012 to June 2022.

The literature in cited journals constitutes the knowledge base of the references, and the research area of highly cited journals is an active interest or emerging area. We used CiteSpace to map and overview literature co-citation relationships in the field of journal research, with the citing journal map on the left and the cited journal map on the right. Notably, the two colored paths shown in **Figure 5** represent the reference relationships for the highly active research areas. Given the correlation between color and quantity, the classification of journals with the highest number of papers was represented by the red path. The knowledge-based research fields of AI application in detecting and grading DR in the past decade consist of engineering technology, computer, medicine, medical imaging, ophthalmology, and biology, which constitute the hot subjects involved in the research frontier, including systems, mathematics, neurology, information science, and ophthalmology.

The top 10 subject categories of the citing journals and the cited journals/proceedings regarding citations are presented in **Tables 3**, **4**. The most common areas of the study cited in journals included engineering technology/biology. The discipline with the most involvement in the cited journals was classified as engineering technology/ophthalmology/biology.

**Table 3:** Top 10 citing journals of publications on the use of AI in DR from January to June 2012.

Rank	Citing journals	Research fields	Counts	Journal impact factor 2021
1	IEEE Access	Engineering Technology/Computer: Information Systems	46	3.476
2	Computer Methods and Programs in Biomedicine	Engineering Technology/Computer: Interdisciplinary Applications	20	7.027
3	Scientific Reports	Comprehensive journal	20	4.996
4	Translational Vision Science & Technology	Medicine/Ophthalmology	20	3.048
5	Computers in Biology and Medicine	Engineering Technology/Biology	19	6.698
6	PLOS ONE	Comprehensive journal	18	3.752
7	Journal of Medical Imaging and Health Information	Medical Imaging/Biology	18	0
8	Journal of Medical Systems	Medicine	15	4.920
9	Sensors	Engineering Technology	15	3.847
10	Biomedical Optics Express	Ophthalmology/Engineering Technology/Biology	14	3.562

AI, artificial intelligence; DR, diabetic retinopathy; IEEE, Institute of Electrical and Electronics Engineers.

**Table 4:** Top 10 cited journals of publications on the use of AI in DR from January 2012 to June 2022.

Rank	Cited journals	Research fields	Counts	Journal impact factor 2021
1	IEEE Access	Engineering Technology/Computer: Information System	216	3.476
2	Scientific Reports	Comprehensive journal	204	4.996
3	PLOS ONE	Comprehensive journal	119	3.752
4	Translational Vision Science & Technology	Medicine/Ophthalmology	117	3.048
5	Biomedical Optics Express	Ophthalmology/Engineering Technology/Biology	109	3.562
6	Computers in Biology and Medicine	Engineering Technology/Biology	93	6.698
7	Multimedia Tools and Applications	Computer Science/Engineering Technology	92	2.577
8	Biomedical Signal Processing and Control	Engineering Technology/Biology	84	5.076
9	Sensors	Engineering Technology	84	3.847
10	Computer Methods and Programs in Biomedicine	Engineering Technology/Computer: Interdisciplinary Applications	82	7.027

AI, artificial intelligence; DR, diabetic retinopathy; IEEE, Institute of Electrical and Electronics Engineers.

### Keywords

**Figure 6:**
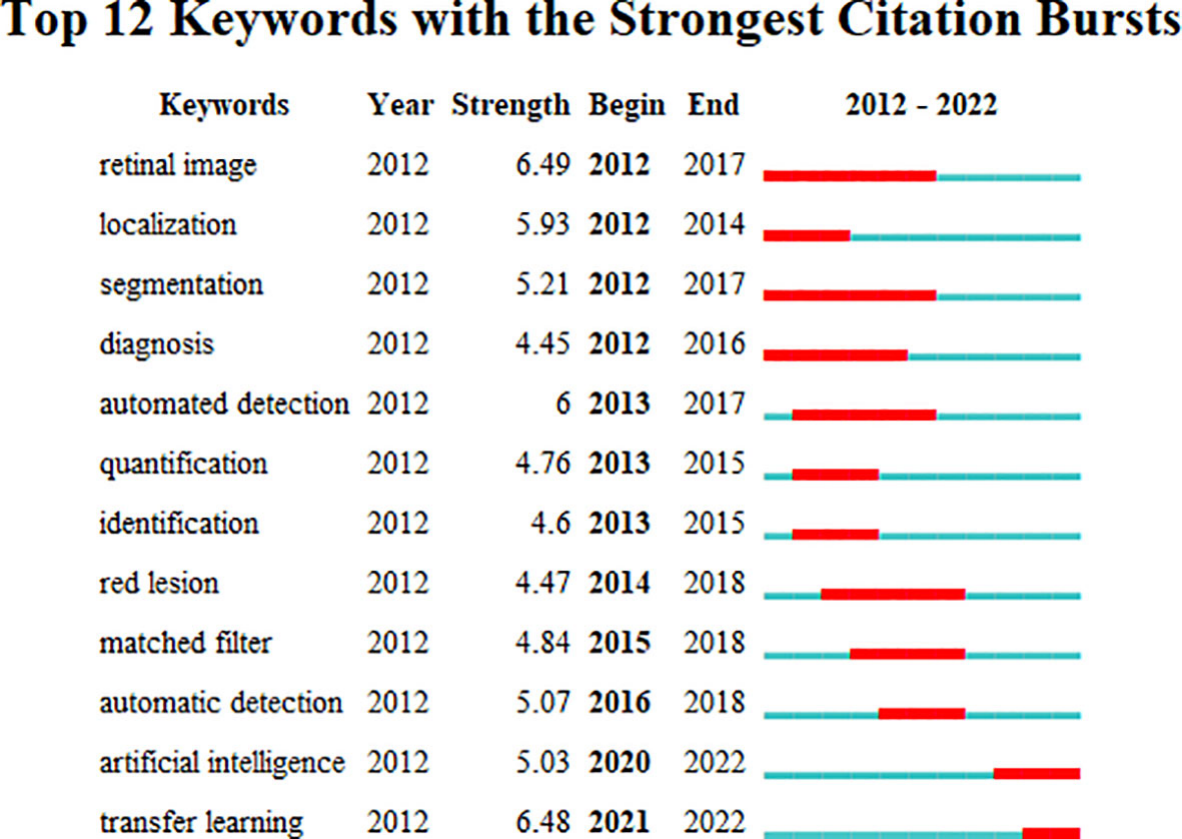
Keywords with the strongest citation bursts for publications on the use of artificial intelligence in diabetic retinopathy from January 2012 to June 2022.

The emerging keywords developed over time were analyzed based on the keyword co-occurrence cooperative network analysis chart, in order to better understand the application of AI in the field of DR diagnosis and gradation in the past decade, reflecting the shift in research hotspots. CiteSpace’s default setting was replaced by the following modes: “Year Per Slice” = 1, “Top N%” = 10.0%, and “Minimum Duration” = 1. The results are shown in **Figure 6**. The burst detection analysis can detect great changes in the number of references in a certain period to determine the fading or rising of a certain subject word or keyword. The emerging keywords for the investigated timeline are depicted as red squares in **Figure 6**. The burst keywords from January 2012 to June 2021 included retinal image (2012–2017), localization (2012–2014), segmentation (2012–2017), diagnosis (2012–2016), quantification (2013–2015), identification (2013–2015), automated detection (2013–2017), red leision (2014–2018), matched filter (2015–2018), automatic detection (2016–2018), artificial intelligence (2020–2022), and transfer learning (2021–2022).

### Citing articles and references

**Figure 7:**
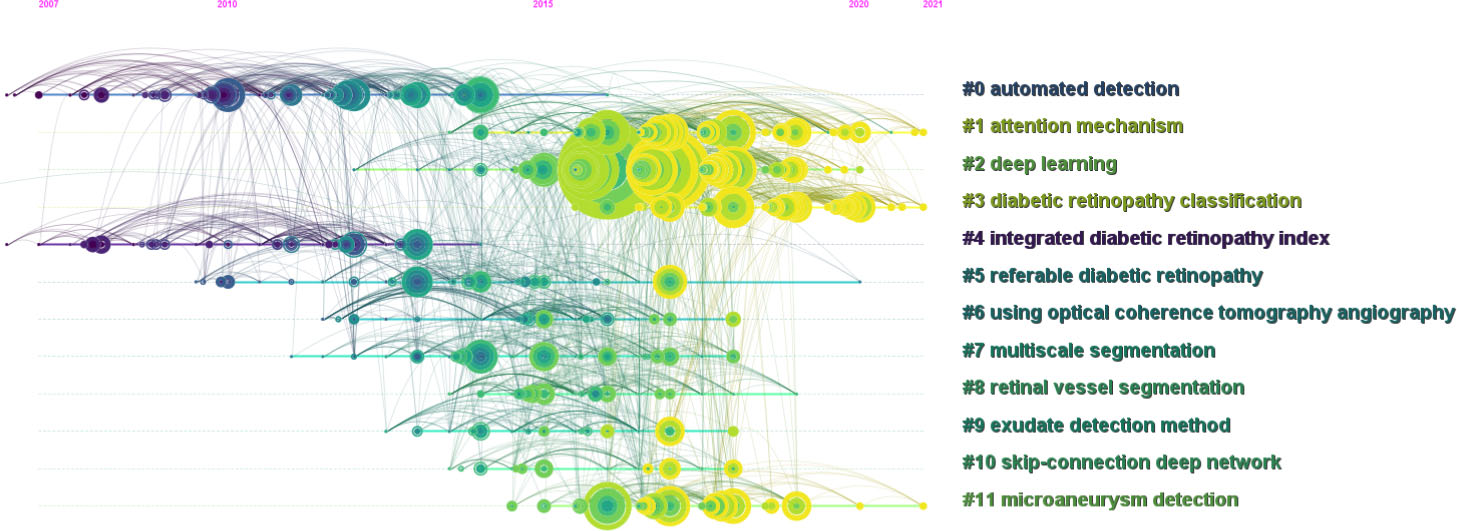
Co-cited reference timeline map of publications on the use of artificial intelligence in diabetic retinopathy from January 2012 to June 2022.

The cited articles were closely related to the research topics. Therefore, a knowledge base for using AI in DR research can be efficiently established through co-citation analysis of cited references. We created a co-citation reference timeline map to identify research topics. **Figure 7** summarizes and presents research topics in the form of clusters, with the size of the clusters listed on the right. The nodes on the timeline in the figure represent the time at which a document was first cited. Furthermore, the node’s size represents the accumulated times the study was cited. The larger the size, the more frequently the document was mentioned. The top label “#0 automated detection” was obtained in the topic generation phase from 2007 to 2014. **Table 5** presents the top 10 citing literature from “times cited in all databases” among the relevant literature based on the application of AI in DR. It was determined that AI techniques could be promising for the application of AI in the diagnosis and grading of DR. However, there were still some limitations when it comes to clinical applications.

**Table 5:** Top 10 cited articles on the use of AI in DR from January 2012 to June 2022.

Rank	Title of citing documents	DOI	Times cited	Interpretation of the findings	Research limitations
1	Development and Validation of a Deep Learning Algorithm for Detection of Diabetic Retinopathy in Retinal Fundus Photographs (6)	10.1001/ jama.2016.17216	2803	Deep machine learning-based algorithms are highly sensitive and specific for the detection of actionable DR.	1. Subtle differences in the images were difficult to explain. 2. The algorithm only showed the grade of the lesion, but not the exact lesion. 3. The scope of the verified data is limited. 4. Only DR and DME can be identified. 5. There are still open questions for further clinical use remaining to be investigated.
2	Development and Validation of a Deep Learning System for Diabetic Retinopathy and Related Eye Diseases Using Retinal Images From Multiethnic Populations With Diabetes (7)	10.1001/ jama.2017.18152	847	The deep-learning system for evaluating retinal images of multi-ethnic diabetic patients has high sensitivity and specificity in identifying DR and associated ocular diseases.	1. The algorithm only showed the grade of the lesion, but not the exact lesion. 2. The clinical diagnostic criteria are different. 3. OCT-assisted diagnosis is still needed in the diagnosis process.
3	Automated Identification of Diabetic Retinopathy Using Deep Learning (13)	10.1016/j.ophtha.2017.02.008	520	A data-driven DL algorithm was developed and evaluated as a novel diagnostic tool for automated DR detection.	Further advice from ophthalmologists or professionals for using the proposed approach and resolving the real-life scenarios is still needed.
4	Improved Automated Detection of Diabetic Retinopathy on a Publicly Available Dataset Through Integration of Deep Learning (14)	10.1167/iovs.16-19964	446	A deep-learning enhanced algorithm for the automated detection of DR achieves significantly better performance.	1. This study is underpowered to determine the detection performance of isolated PDR without ME. 2. Lack of flexibility as a practical clinical diagnostic application. 3. DR and ME prevalence and severity may be underestimated in this dataset, and a different reference standard could lead to differences in a device’s measured algorithmic performance.
5	Pivotal trial of an autonomous AI-based diagnostic system for detection of diabetic retinopathy in primary care offices (15)	10.1038/s41746-018-0040-6	412	The first FDA-authorized autonomous AI diagnostic system can detect more than mild DR and diabetic macular edema, considering affordability, quality, and accessibility.	1. Limitations of the spectrum of disease. 2. OCT-assisted diagnosis is still needed in the diagnostic process. 3. The sensitivity of the AI system was lower than that of a similar system.
6	ReLayNet: retinal layer and fluid segmentation of macular optical coherence tomography using fully convolutional networks (16)	10.1364/BOE.8.003627	285	A new fully convolutional deep architecture, termed ReLayNet, was proposed for end-to-end segmentation of retinal layers and fluid masses in eye OCT scans.	1. OCT-assisted diagnosis is still needed in the diagnostic process. 2. The scope of the verified data is limited. 3. There are still open questions for further clinical use that remain to be investigated.
7	Exudate-based diabetic macular edema detection in fundus images using publicly available datasets (17)	10.1016/j.media.2011.07.004	199	This study presented a new exudate-based automatic system for detecting DME using non-stereo fundus images	A competitive DR-screening system that can transparently diagnose the disease state remains to be created.
8	TeleOphta: Machine learning and image processing methods for teleophthalmology (18)	10.1016/j.irbm.2013.01.010	198	A complete prototype for the automatic detection of normal examinations on a teleophthalmology network for DR screening is presented.	1. The effectiveness of the developed method is yet to be confirmed. 2. The interpretability of the existing intelligent diagnostic system was not investigated. 3. The system sensitivity is not chosen concerning future clinical trials.
9	Automated Analysis of Retinal Images for Detection of Referable Diabetic Retinopathy (19)	10.1001/jamaophthalmol.2013.1743	193	Computer analysis of retinal photographs for DR and automatic detection of RDR can be safely carried out in DR screening, and early treatment can reduce vision loss.	1.Clinical classification criteria have not been unified. 2.Retinal images are not stereoscopic and may not reveal possible pathogenic factor like clear fluids. 3.The data used in the study has limitations: The IDP only compared readings from retinal specialists and did not consider readings from post-mydriatic retinal examinations.
10	Deep image mining for diabetic retinopathy screening (20)	10.1016/j.media.2017.04.012	183	Deep image mining is based on DL for the automatic detection of referable DR and for the automatic detection of lesions related to DR.	1. The algorithm only displayed the lesion grade and did not count the actual DR lesions 2. Manual assistance in diagnosis is still needed.

AI, artificial intelligence; DR, diabetic retinopathy; OCT, optical coherence tomography; DL, deep learning; FDA, United States Food and Drug Administration; DME, diabetic macular edema; DOI, digital object identifier.

## Discussion

### Principal results

Based on the above outcomes, the amount of published literature on DR AI detection and intelligent diagnosis has significantly increased over the past 4 years. This indicates that, as the demand for screening, diagnosis, classification, and follow-up of DR increases, so has the interest in the application of AI in DR.

The development of artificial intelligence takes the clinical practice of ophthalmology as a research frontier (21), with DR being the most studied eye disease (22). DL algorithms were verified to be highly sensitive and specific (6, 7) in detecting and classifying DR and related eye diseases, which is a promising research field. Regarding the amount of national literature, China has the largest number of articles; however, a few articles have been highly cited. The United States has the highest centrality and H-index, indicating that it is in the lead in this research field. Moreover, the United Kingdom, India, and Pakistan have considerable centrality and influence. The top three research institutions by the number of publications were the United States, China, and Singapore. Regarding the H-index, Oregon Health and Science University in the United States and the National University of Sciences and Technology in Pakistan strongly influence this area. The focus of AI research has changed from upgrading the accuracy and efficiency of DR detection and classification to research on DR diagnostic systems. AI is applied to the diagnosis and classification of DR, which is usually performed based on fundus photography and optical coherence tomography (OCT) analysis, using retinal vessel segmentation and directional local contrast to detect lesions, including microaneurysms and hard exudation. Furthermore, clustering timelines based on emerging keywords can identify active areas of interest, research frontiers, and commonly cited references of AI in DR diagnosis. In 2018, the United States Food and Drug Administration approved an AI system that could detect referential DR from retinal photographs. This is the first independent diagnostic system approved in the medical field (13). In addition, this indicates that AI DR intelligent diagnosis systems have broad prospects in clinical applications and are expected to promote the reform of clinical diagnosis systems.

### Hot knowledge base in the start period

Through clustering analysis of the results of the co-cited articles, it can be easily determined that “automated detection” and “intergrated DR index”, were the most important knowledge bases in the early stages of studies. This illustrates that the focal point of this study was to improve the efficiency and accuracy of DR lesion detection and classification.

Automated monitoring and screening for DR could significantly reduce manpower and time. Meanwhile, early treatment can reduce visual impairment caused by disease progression. Therefore, automatic detection of DR has been a hot topic in this field. The automatic detection technology for DR has been constantly improving. In the early stage, the intelligent recognition and diagnosis of DR-related lesions in fundus images were mainly detected by machine learning(ML). During this period, a variety of ML tools for DR screening were developed, such as decision tree, support vector machine (SVM), artificial neural network (ANN), Bayesian classifier, and so on. However, the recognition efficiency of traditional ML is limited, and sometimes manual confirmation is needed. Therefore, some scholars have tried to integrate various algorithms by adding ensemble learning algorithms on the basis of ML, so that the computer can identify the lesions related to DR through different strategies. Antal et al. (23) established a new ensemble-based system to detect microaneurysms and grade DR, and achieved an area under the curve (AUC) of 0.875. Since 2016, due to the gradual application of DL in automatic DR recognition, the efficiency and accuracy of automatic detection have been greatly improved.

Previous studies have shown that it is difficult to distinguish fundus images from normal images in patients with DR by using traditional measurement methods based on texture features. In contrast, the advantage of the integrated index is that it only examines the value of one composite index, which represents the degree of deviation from the normal, to make a correct diagnosis. The latter can improve the accuracy of lesion detection and disease classification and significantly facilitate the work of clinicians. Acharya et al. (24) developed an integrated DR index (IDRI) comprising textural features that can be used to diagnose normal and different stages of DR.

### Research hotspots

The emerging keywords between 2012 and 2017 were “retinal image,” “localization,’’ “segmentation,”and “diagnosis.” Therefore, this indicates that the initial research focused on the study of intelligent algorithms used to segment and localize lesions on retinal images to assist in the diagnosis of DR. Retinal images provide valuable information related to the human eye; therefore, assessment of the characteristics of retinal blood vessels is important for disease diagnosis based on vascular pathology (25). A fundus image database is an important training and testing object for AI recognition and diagnostic systems. Franklin et al. (26) developed a novel automatic blood vessel segmentation method for fundus images that separated each image pixel into blood and non-blood vessels for automatic recognition of microaneurysms in retinal images. The detection of retinal microaneurysms using a multilayer perceptron neural network is helpful for ophthalmologists in diagnosis, and follow-up DR. Diagnostic localization on fundus images and OCT were as follows: exudates (17), optic disk (27), and dark and bright lesions (28). The efficiency of machine recognition during this period was limited and sometimes required manual confirmation, which differed from the current intelligent diagnosis that depended on DL (29). The traditional ML recognition system is commonly used as an auxiliary tool for clinicians to screen and diagnose (30).

The emerging keywords between 2013 and 2017 were “quantification,” “identification,’’ and “automated detection.” Therefore, this indicates that quantization processing of image features is gradually applied in DR automatic recognition. Finding and counting lesions, including microaneurysms, or exudation, on retinal images is crucial for the clinical diagnosis of DR. However, it is time-consuming and can be affected by human error. Accordingly, automatic layer detection technology for the human retina has been developed to improve its efficiency. Traditional diagnosis relies on experience and tends to supervisor error; therefore, an objective standard is required to measure the result of the diagnosis; that is, the automatic quantification of blood vessels in human retina is the guarantee of reducing the subjective error of intelligent diagnosis and enhancing accuracy (31). Wu et al. (32) developed a computer-aided quantification framework for the automatic detection of exudates and microaneurysms and compared the morphological characteristics of moderate and severe non-proliferative DR. The results showed that computer-aided quantification of DR could be a practical method for clinicians to better study DR.

The emerging keywords between 2015 and 2018 were “red lesion,” and “matched filter.” Therefore, this indicates that as an essential biomarker for the early diagnosis of DR, the detection of red lesions, including microaneurysms and hemorrhages, has become an important research direction in the intelligent diagnosis of DR. A matched filter is a segmentation algorithm that can be used to perform vessel extraction and detect microaneurysms. It is vital to detect red lesions, especially microaneurysms, from color fundus images for the early diagnosis of DR. However, accurate automatic detection of microaneurysms on color retinal images is still a challenging problem. One of the important solutions is to screen microaneurysms in images by vessel extraction using a multiscale matched filter. Filter-based microaneurysm detection algorithms are examples of vessel extraction (33–36). In addition to microaneurysms, researchers are also actively exploring the association of other biomarkers with DR to provide new directions for the intelligent diagnosis and staging of DR. For example, Niu et al. (37) highlighted that the area and number of hard exudate and hyperreflective lesions were related to DR staging. Cheung et al. (38) investigated the relationship between the geometric variables of retinal blood vessels measured from retinal photographs and the 6-year incidence and progression of DR.

The emerging keywords between 2016 and 2018 is “automatic detection”. In this period, DL algorithms, including the application of convolutional neural networks (CNN) in DR, drive these advances and transformations. Deep learning(DL) is a set of computational methods that allow algorithms to program themselves by learning from a large number of examples. These examples show only the desired behavior, not explicit calculation rules.(6) Compared with the traditional supervised learning, DL greatly improves the accuracy of automatic detection while reducing the workload, and has obvious advantages. In 2016, Abràmoff et al.(14) demonstrated that integrating convolutional neural networks on top of existing pathology-based diabetic retinopathy significantly improved the recognition performance of referable diabetic retinopathy compared to the same algorithm without DL techniques. CNN is one of the renowned DL algorithms in image-related applications (39). It has made great advances in the field of computer vision research by constantly revising and self-learning to complete the task (40, 41). In 2016, Gulshan et al. (6) created an algorithm to automatically detect DR and DME based on retinal fundus photos using deep CNN. This study demonstrated that deep ML based algorithms have high sensitivity and specificity for detecting referable diabetic retinopathy(RDR) in the evaluation of retinal fundus photos of adult diabetic patients. Many studies have shown that CNN has become a major tool for DR screening, staging and prediction, and possesses extensive application prospects (42, 43).

The emerging keywords between 2020 and 2022 were “Artificial intelligence” and “transfer learning.” This indicates that artificial intelligence including transfer learning is a hot topic in recent research. Although DL models provide automatic feature extraction and classification, it still needs a large number of annotated data sets to start training such intelligent models. The model based on transfer learning is widely used by researchers to overcome the problem of insufficient annotation data and computational overhead. Le et al. (44) constructed a retrained CNN for DR classification based on OCTA images with 83.76% sensitivity and 90.82% specificity *via* transfer learning.

AI is an intelligent system mainly based on DL, which has developed rapidly in recent years. This research field involves the deep integration of multi-disciplinary knowledge and technology, and requires the cooperation and sharing of multi-disciplinary technical resources. At present, there are still problems such as data standardization, insufficient clinical verification and products waiting to come into service. Although opportunities and challenges coexist in the research of AI-assisted DR screening, with the gradual deepening of the study and the joint efforts of relevant interdisciplinary researchers, the clinical application of AI-assisted diagnosis of DR in ophthalmology is expected to make greater progress.

### Limitations in research of AI in DR

The constraints of AI in diagnosing ophthalmic diseases were classified into the following five categories after summarizing the limitations of the top 10 cited articles in **Table 5**: 1) the design of intelligent diagnostic systems is complicated by complex clinical conditions and subjective evaluation criteria; 2) the sample size of the AI training model is relatively limited, and the actual validity requires confirmation; 3) the existing auxiliary diagnostic system is incapable of independent diagnosis, and the specific clinical application requires the advice of ophthalmologists or professionals; 4) inconsistent clinical reference standards may lead to differences in the performance of intelligent algorithms; and 5) the interpretability of the existing intelligent diagnostic system is unsatisfactory.

The following are the recommendations made to promote the application of AI technology in the diagnosis and intelligent classification of DR. First, in order to develop more robust and available diagnostic systems, more types and larger data sets are required. A unified high-quality DR image database and continuous optimization and expansion of the database are required to meet the diagnostic needs of DR to solve the problem of the number of samples. For example, establishing a database that can integrate data from different sources and address fragmented data could be useful for further diabetic research (45). Second, the research scope is required to be comprised of more common disease types, such as glaucoma and age-related macular degeneration (46, 47). Third, a unified output standard for DR disease diagnosis is required to guarantee the versatility of various systems. Fourth, more ophthalmologists in various professional levels should be involved in the dataset’s screening phase and the algorithm’s examination stage to obtain a clinical diagnosis. Fifth, the legal status of AI diagnosis should be guaranteed, and when the technology is advanced, AI can be approved for independent diagnosis. Finally, relevant professionals should strengthen technical research and enhance technology visualization to further improve the usage rate of AI technology.

### Limitations in this study

There are a few latent limitations in this study. Firstly, given that the research method of this study is to analyze the previous literature, the prospective of the study may not be accurate enough. Meanwhile, there is a time period from research to publication. The published articles are often out of sync with the actual research time. Secondly, only English literature in the WoSCC database, a prominent academic database, was analyzed. Literature in other databases or other languages was not included considering the impracticability of fusing and analyzing data from different databases or data in different languages at the same time. Thirdly, the study mainly analyzed the application of AI technology in onefold DR cases, and did not analyze the application of AI in multiple retinal diseases.

### Conclusion

Recently, intelligent algorithm training based on image analysis has attracted increasing attention. AI’s application in the DR screening and diagnosis is being studied worldwide. Particularly, the United States currently has the greatest clout in this research field. The application of AI in the screening and diagnosis of DR has significantly altered the clinical setting of ophthalmologists and patients. These technologies provide more rigorous, faster, and remote diagnostic services. However, these methods have certain limitations. For example, the sample size of the AI training model was limited, and the actual effectiveness of its clinical use requires confirmation. In addition, existing models can only be used as auxiliary diagnostic tools, and ophthalmologists or professionals still need to provide suggestions for clinical application. Furthermore, most contemporary studies are still at the system development and testing stage, and a sophisticated diagnostic system is yet to be developed. Initial research focused on the analysis of intelligent algorithms used to localize or recognize lesions on fundus images to assist in the diagnosis of DR. Currently, the focus of research has shifted from upgrading the accuracy and efficiency of DR lesion detection and DR classification to research on DR diagnostic systems. Therefore, acquiring more national and ethnic origins and merging more complex ophthalmic data is a necessity in training and testing the algorithm in order to address the existing limitations. However, it is a challenging task. In addition to computer engineering specialists who mainly develop algorithms, ophthalmologists in various professional levels are also needed to be involved in this research.

## Data availability statement

The original contributions presented in the study are included in the article/supplementary material. Further inquiries can be directed to the corresponding authors.

## Author contributions

RW and GZ conceived and designed the analysis, performed the analysis, and wrote the manuscript; KL and WL revised the manuscript; ZX, YH, and WY designed the research, acquired the article information, and revised the manuscript. All authors contributed to the article and approved the submitted version.

## Funding

Supported by Shenzhen Fund for Guangdong Provincial High-level Clinical Key Specialties (SZGSP014) and Sanming Project of Medicine in Shenzhen (SZSM202011015).

## Conflict of interest

The authors declare that the research was conducted in the absence of any commercial or financial relationships that could be construed as a potential conflict of interest.

## Publisher’s note

All claims expressed in this article are solely those of the authors and do not necessarily represent those of their affiliated organizations, or those of the publisher, the editors and the reviewers. Any product that may be evaluated in this article, or claim that may be made by its manufacturer, is not guaranteed or endorsed by the publisher.
